# Attention deficit hyperactivity disorder (ADHD) as a risk factor for infection with COVID-19

**DOI:** 10.1192/j.eurpsy.2021.300

**Published:** 2021-08-13

**Keywords:** ADHD, Adult, risk, COVID-19

## Abstract

**Introduction:**

ADHD limits the ability to comply with Covid-19 prevention recommendations. We hypothesized that ADHD constitutes a risk factor for Covid-19 infection and that pharmacotherapy may lower that risk.

**Objectives:**

To test our hypothesis we studied the data of all patients admitted to (N=14,022) Leumit Health Services in Israel between February 1^st^ - April 30th, 2020, who underwent at least one Covid-19 test.

**Methods:**

Data were collected from the electronic health records. Purchasing consecutively at least 3 ADHD-medication-prescriptions during past year was considered drug-treatment.

**Results:**

1,416 (10.1%) subjects (aged 2 months - 103 years) were Covid-19-positive.They were significantly younger, and had higher rates of ADHD (adjOR 1.58 (95%CI; 1.27-1.96, p<0·001) than Covid-19-negative subjects. The risk for Covid-19-Positive was higher in untreated-ADHD subjects compared to non-ADHD subjects [crudeOR 1.61 (95%CI 1.36-1.89, p<0.001)], while no higher risk was detected in treated ones [crudeOR 1.07 (95% CI 0·78-1.48 p=0.65)].

**Conclusions:**

Untreated ADHD seems to constitute a risk factor for Covid-19 infection while drug-treatment ameliorates this effect.

**Disclosure:**

No significant relationships.

